# Tester selection for combining ability estimation of storage root yield and sweetpotato virus disease in sweetpotato breeding

**DOI:** 10.1038/s41598-025-88609-w

**Published:** 2025-02-10

**Authors:** Jolien Swanckaert, Iara Gonçalves dos Santos, Saulo F. S. Chaves, Reuben Ssali, Robert O. M. Mwanga, Camila Ferreira Azevedo, Thiago O. Mendes, Bert De Boeck, Raul Eyzaguirre, Mercy Kitavi, Dorcus C. Gemenet, Maria Andrade, Wolfgang J. Grüneberg, Hannele Lindqvist-Kreuze, G. Craig Yencho, Hugo Campos, Guilherme da Silva Pereira

**Affiliations:** 1 International Potato Center (CIP), Kampala, Uganda; 2 Federal University of Viçosa (UFV), Viçosa, Brazil; 3 International Potato Center (CIP), ILRI Campus, Nairobi, Kenya; 4 International Potato Center (CIP), Lima, Peru; 5 Michigan State University (MSU), East Lansing, USA; 6 International Maize and Wheat Improvement Center (CIMMYT), Nairobi, Kenya; 7 International Potato Center (CIP), Maputo, Mozambique; 8 North Carolina State University (NCSU), Raleigh, USA

**Keywords:** *Ipomoea batatas*, Heterosis, Hybrids, GCA, Breeding optimization, Plant breeding, Plant genetics

## Abstract

General combining ability (GCA) is the major selection criterion for new sweetpotato (*Ipomoea batatas*) parents in a reciprocal recurrent selection (RRS) scheme. Here we aimed to estimate GCA and specific combining ability (SCA) by using 16 potential testers involved in an 8 × 8 partial diallel and propose a procedure to identify testers in sweetpotato breeding. Data on storage root yield in tons per hectare (rytha), and sweetpotato virus disease (vir2) from 64 families (1,913 clones) were collected in five trials at two locations in Uganda. The estimates of the female GCA accounted for the largest additive genetic variation for storage root yield compared to the male GCA for both traits. Mid-parent heterosis ranged from − 6.2 to 7% for rytha, and − 1.1 to 1.3% for vir2 in the progeny families. A stepwise procedure to identify testers top-ranked ‘NASPOT 7’ as a dual tester for both traits. Besides this parent, ‘Ejumula’ and ‘NASPOT 10 O’ for rytha, and ‘NASPOT 1’, ‘NK259L’, ‘SPK004’, and ‘NASPOT 11’ for vir2 are particularly suitable as respective single-trait testers. Testers are important in many plant breeding programs to enhance efficiency of RRS, and thus other crop species might benefit from the strategy and methods applied herein.

**Supplementary Information:**

The online version contains supplementary material available at 10.1038/s41598-025-88609-w.

## Introduction

Sweetpotato [*Ipomoea batatas* (L.) Lam. (2n = 6x = 90)] is a staple food in some countries in Africa and has been widely cultivated all over the world^1^. The crop plays a role in nutritional security due to its levels of carbohydrates, vitamins, and micronutrients^2^. However, its nutritional potential is sometimes hampered by low storage root yield or susceptibility to pests and diseases. To enhance breeding efficiency, breeders have introduced reciprocal recurrent selection (RRS) in sweetpotato. The use of two genetically diverged pools to exploit heterosis and offspring testing could potentially increase the proportion of complementary loci in hybrid populations^3^. Intra-pool crosses are also performed to improve the genetic pools separately, increasing divergence in the long term. The difference in allele frequency between pools leads to increased heterozygosity in the inter-pool F_1_ sweetpotato hybrids. As such, the F_1_ hybrids display heterosis due to dominance and some types of epistasis, even if the difference in allele frequency is not due to previous RRS. In maize, where the hybrid breeding concepts were first introduced, breeders rely on partially or completely inbred lines to reproduce the heterotic parents stably. Since sweetpotato can be clonally propagated, hybrid breeding can simply utilize heterosis via RRS with moderate inbreeding by crossing relatives.

The selection of new parents is a crucial step in any plant breeding program. A key performance indicator such as average cycle time can be used to evaluate a breeding program by considering the average age of parents in the crossing block^4^[Chap. 9]. In an RRS, the pathway for variety development is separated but linked to population improvement. The information from inter-pool crosses increases the likelihood of selecting complementary parents within each pool and getting a superior offsprings. In the population improvement, intra-pool crosses of selected individuals aim to maintain in the population alleles that confer high performance and complement well with alleles from the other population while discarding unfavorable or lethal alleles. Such a two-part program (population improvement and variety development) is supposed to deliver larger genetic gains than a conventional program for the same investment in both inbred^5^ and outbred crops^6^.

Combining ability in crosses can be partitioned into components, comprising general (GCA) and specific (SCA) combining abilities^2^. GCA measures the parent’s breeding value in a set of crosses and can be used to help determine the medium- and long-term response to selection when selecting new parents. Increased yields due to hybrid vigor can be captured in the SCA, which includes the non-additive effects^7^. For sweetpotato, GCA effects have been reported as the predominant effect and larger than SCA^8^. A large GCA relative to SCA makes breeding easier and more efficient.

The conventional approach to estimate GCA and SCA is through phenotyping the offspring clones of a set of structured crosses from full or partial diallel. In sweetpotato, bi-parental crosses generate true seeds, which are germinated, and the resulting plants are vegetatively multiplied and observed in clonal plots over several environments. As the true seeds typically come from outbred parents, each seed is genetically unique. When more parents are considered in the genetic design, a larger set of offspring clones and field trials are needed. Owing to relatively high cross incompatibility in sweetpotato, there is a risk of underrepresenting parents with difficulties in flowering and producing seeds.

Another method to estimate GCA is line × tester analysis, also called topcross^9^. Testers are commonly used in hybrid breeding schemes and breeding synthetics^10^. Most hybrid breeding schemes are based on at least two pools, which are preferably mutually heterotic, or are developed over time to become mutually heterotic. In sweetpotato, large genetic gains have been reported after one complete RRS cycle associated with pronounced heterosis increments in three applied orange-fleshed sweetpotato hybrid populations aiming at four target product profiles^11^. On basis of stochastic simulations, there are opinions that hybrid schemes based on two pools might not benefit autopolyploid crops, such as hexaploid sweetpotato^12^. However, it should be noted that older simulation studies also recommended not to work with two pools in diploid hybrid breeding such as maize and that gene-pools should be merged^13^, which proved to be misleading in case of maize^14^. In this study, it is hypothesized that testers can assess the suitability of potential parents and subsequently define the direction of the offspring clones (that serve as parents in the next generation), and that testers are expected to enhance the population mean and maximize genetic gain if properly chosen.

Here, we use the Mwanga diversity panel (MDP), which was originated from crossings between 16 clones (8 × 8 diallel) from two pseudo-heterotic groups^15,16^. Among these clones, there are breeding materials, landraces and commercial cultivars from different countries (mostly Uganda) that hold commercially interesting traits, like resistance to pests and diseases, high root yield, and appropriate root shape and color^16^. The trials were spread across multiple environments in Uganda and were fit to estimate the GCA, SCA and genetic parameters from both parents and offspring^17^. The objectives of this study were (i) to estimate genetic values for parents and offspring, genetic variances, and heritability for storage root yield and sweetpotato virus disease (SPVD) across environments, and (ii) to identify potential testers using a three-step approach.

## Materials and methods

### Plant material and experimental design

A total of 16 parents coming from two pools were used in this study. The two pools, identified here as A (8 male varieties) and B (8 female varieties) (Suppl. Table S1), were established based on simple sequence repeat (SSR) markers^18^. The source of pollen, male parents, was assigned as reported by Swanckaert et al.^19^. The bi-parental crosses between B × A (8 × 8 crosses) resulted in 64 families that were evaluated in clonal plots (~30 offspring clones per cross) along with the 16 parents.

Trials were planted using a row-column design in which the sixteen parents were randomized between the unreplicated clonal plots using a grid comprising two check clones. The checks were ‘NASPOT 11’, an SPVD resistant clone from pool B, and ‘Ejumula’, an SPVD susceptible clone from pool A. The plot spacing was 0.3 m between plants within a row and 1 m between rows.

Trials were conducted in Namulonge at the National Agricultural Research Organization (NARO) research station in Uganda under high SPVD pressure (0°31’22” N, 32°38’09"E, 1,137 m above sea level, m.a.s.l.), and in Serere at the National Semi-Arid Resources Research Institute (NaSARRI) (1°32’N, 33°27’E, 1,100 m.a.s.l.). The two locations, Namulonge and Serere, are situated, respectively, in two major agro-ecologies in Uganda, namely (i) the warm, moist, tall grassland savanna where SPVD pressure is severe, and (ii) the warm, subhumid short grassland savanna where sweetpotato weevil (SPW) and drought are observed^17^. The trials were conducted in 2018 seasons A and B, and 2019 season A (five environments in total). Season A corresponded to the main rainy season (planted in April, harvested in September), whereas season B corresponded to the less reliable shorter rainy season (planted in September, harvested in March). Climate data were downloaded and sorted using the R packages EnvRtype^20^ and geodata^21^ (Suppl. Fig. S1). Soil samples were collected from the experimental sites and processed for standard soil chemical and physical analyses (Suppl. Fig. S2). We computed the storage root yield (tons per ha – rytha), and the incidence of SPVD symptoms (scores 1–9, with lower values indicating fewer symptoms, an indirect measure of resistance – vir2) (Suppl. File S1).

The sweetpotato germplasm (Suppl. Table S1), composed of six Ugandan landraces, five bred varieties, one Kenyan landrance, and two introductions (‘Huarmeyano’ and ‘Resisto’), was used by NARO for sweetpotato improvement in previous years^16^. The two introductions are in the International Potato Center (CIP) gene bank for free distribution. All the sweetpotato germplasm was donated by NARO to CIP, and land for the trials was made available free of charge based on an agreement on technical cooperation in research and training between the Government of the Republic of Uganda and CIP signed in 1988.

### Statistical analyses

Phenotypic data were analyzed in a two-stage approach. In the first stage, spatial trends were modeled using the Spatial Analysis of field Trials with Splines (SpATS) mixed model^22,23^ using the *spl2D* function of the R package sommer v. 4.1.2^24^ according to the model as follows:

1 $$\:\begin{array}{c}\varvec{y}=\varvec{X\beta}\:+\varvec{Ws}+\varvec{e}\end{array}$$

where $\varvec{y}$ refers to the vector of phenotypic observations of storage root yield or SPVD resistance for the 16 parents and its progenies in the 64 families, $\varvec{\beta}$ is the vector of fixed effects of the intercept, $\mu$, and checks, and $\varvec{X}$ is the associated design matrix; $\varvec{s}$ is the vector containing the fixed (unpenalized) and random (penalized) components of the smooth spatial surface and the mutually independent sub-vectors of row and column effects that have an associated (co)variance matrix $\varvec{S}$, and $\varvec{W}$ is the associated design matrix; and $\varvec{e}$ is the random vector of independent residuals, $\:\varvec{e} \sim N(\varvec{0}, \varvec{I}{\sigma}_{e}^{2})$, where $\varvec{I}$ is an identity matrix and ${\sigma}_{e}^{2}$ is the residual variance. We did not consider the genotype effects, which were confounded within the residuals. By doing so, we removed the design effects, and generated a modified $\varvec{y}$, ${\varvec{y}}^{*}$, consisting of residual plus the intercept to be analyzed in the second step, i.e. ${\varvec{y}}^{*}=\varvec{1}\mu\:+\varvec{e}$.

To estimate traits’ heritability values per environment, we fitted the previous model (Eq. 1) including the genotype effect as random, $\varvec{g} \sim N(\varvec{0}, \varvec{H}{\sigma}_{g}^{2})$, where $\varvec{H}$ is a hybrid relationship matrix combining the genomic information of parents [Suppl. Fig. S3]^16^ and the pedigree information of the population. We built $\varvec{H}$ based on Martini et al.^25^ using the *Hmatrix* function of the R package AGHmatrix^26^ considering ploidy = 6. Using this model, we obtained the genotypes’ best linear unbiased predictions (BLUPs), and computed the generalized heritability^27^, as follows:

2 $$H^{2} = 1 - \frac{{\overline{V} \left( {{{\varDelta}}} \right)}}{{2\sigma_{g}^{2} }}$$

where $\overline{V} \left( {{\varDelta}} \right)$ is the mean variance of a difference between the genotypic BLUPs.

### Testers’ identification

**Fig. 1:**
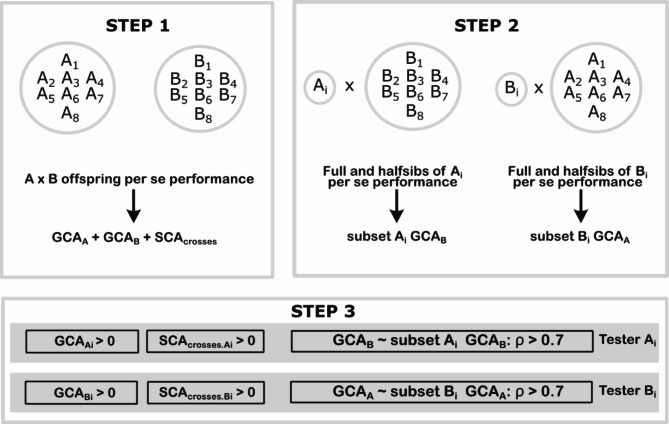
Three-step approach to identify potential testers within pools. In Step 1, GCA effects for males (A_1_, A_2_, …, A_8_) and females (B_1_, B_2_, …, B_8_), and SCA effects for crosses (SCA_crosses_) generated predictions for each family. In Step 2, GCA values for each of the 16 parents (males A_i_ and females B_i_) were estimated based on the set of parents in the opposite pool. Finally, Step 3 identifies potential female or male testers based on GCA, SCA, and correlation ($\rho$) between the GCA obtained in STEP 1 and the GCA estimates from the subset of parent $i$ (STEP 2). SCA_crosses.A_ and SCA_crosses.B_ are SCA estimates of the crosses for male A_i_ and female B_i_, respectively.

Potential testers were identified using a three-step approach (Fig. 1). In Step 1, the averaged GCA effects for males (A) and females (B), and SCA effects for crosses were obtained from the predictions of each environment using the mixed model as follows:

3 $$\:\begin{array}{c}\:\:{\varvec{y}}^{*}=\varvec{1}\mu +{\varvec{Z}}_{A}{\varvec{g}}_{A}+{\varvec{Z}}_{B}{\varvec{g}}_{B}+{\varvec{Z}}_{C}{\varvec{g}}_{C}+\varvec{e}\end{array}$$

where ${\varvec{y}}^{*}$ is the adjusted value obtained in the first stage, $\mu$ is the fixed intercept; ${\varvec{g}}_{A},{\varvec{g}}_{B},{\varvec{g}}_{C}$ are random vectors of GCA for males, GCA for females, and SCA for crosses, respectively, with ${\varvec{g}}_{A} \sim N (\varvec{0},{\varvec{G}}_{A} \sigma_{A}^{2} ),$ ${\varvec{g}}_{B} \sim N(\varvec{0},{\varvec{G}}_{B} \sigma_{B}^{2} )$, and ${\varvec{g}}_{C} \sim N(\varvec{0},{{\varvec{G}}_{A} \otimes {\varvec{G}}_{B}\sigma}_{C}^{2})$, ${\sigma}_{A}^{2},{\sigma}_{B}^{2},{\sigma}_{C}^{2}$ are genetic variances of males (pool A), females (pool B), and crosses (A × B), ${\varvec{Z}}_{A},{\varvec{Z}}_{B},{\varvec{Z}}_{C}$ are the respective incidence matrices, ${\varvec{G}}_{A}$ and ${\varvec{G}}_{B}$ are the subsets of the genomic relationship matrix that contain data only from males and females, respectively; and $\otimes$ is the Kronecker product. GCA and SCA effects significance were assessed using likelihood ratio tests (LRT).

The vector of family predictions, $FP$, was calculated as:

4 $$\:\begin{array}{c}FP=\:{\widehat{\varvec{g}}}_{A}+{\widehat{\varvec{g}}}_{B}+{\widehat{\varvec{g}}}_{C}\end{array}$$

Mid-parent heterosis increments ($MPH$, in percentage) were estimated as follows:

5 $$\:\begin{array}{*{20}c} {MPH_{{\left( \% \right)}} = \frac{{\overline{{F_{1} }} - MP}}{{MP}} \times 100} \\ \end{array}$$

where $\overline{{F_{1} }}$ is the progeny mean, and $MP$ is the mean of the two parents obtained from Eq. 3.

In Step 2, the dataset was subdivided into subsets per parent, thus including all full- and half-sibs. Using these subsets, new GCA values for each of the 16 parents were estimated based on the set of parents in the opposite pool, i.e. each female (or male) had a GCA considering all the males (or females) in the opposite pool. For instance, Wagabolige’s subset has data from all families in which this clone was a female parent, resulting in its GCA estimated from crosses with all males. The new estimates were obtained for each environment according to the following model:

6 $$\:\begin{array}{c}{\varvec{y}}^{*}=\varvec{1}\mu + {\varvec{Z}}_{A\:\text{or}\:B}{\varvec{g}}_{A\:\text{or}\:B}+\varvec{e}\end{array}$$

where ${\varvec{g}}_{A\:\text{or}\:B}$ is the random vector of GCA for males or females, respectively, with ${\varvec{g}}_{A\:\text{or}\:B} \sim N(\varvec{0},{\varvec{G}}_{A\:\text{or}\:B}{\sigma}_{A\:\text{or}\:B}^{2}),$ ${\sigma}_{A\:\text{or}\:B}^{2}$ is the genetic variance of males or females, ${\varvec{Z}}_{A\:\text{or}\:B}$ is the associated incidence matrix, and ${\varvec{G}}_{A\:\text{or}\:B}$ is the genomic relationship matrix of males or females.

In Step 3, a potential tester $i$ was defined when it fulfilled three requirements: (i) positive GCA for parent $i$, (ii) positive SCA averaged over all crosses for parent $i$, (iii) Pearson correlation, $\rho$, between GCA from Step 1 and GCA estimated from the subset of parent $i$ from Step 2 greater than 0.70, a strong correlation value^28^. The idea is that a reliable tester allows a proper estimation of the other parent’s potential. In this case, a high correlation between the parents’ GCA using the complete dataset and using only a subset of families considering a given parent is expected.

## Results

### Performance of parents and offspring, genetic variance, and heritability per environment

**Fig. 2:**
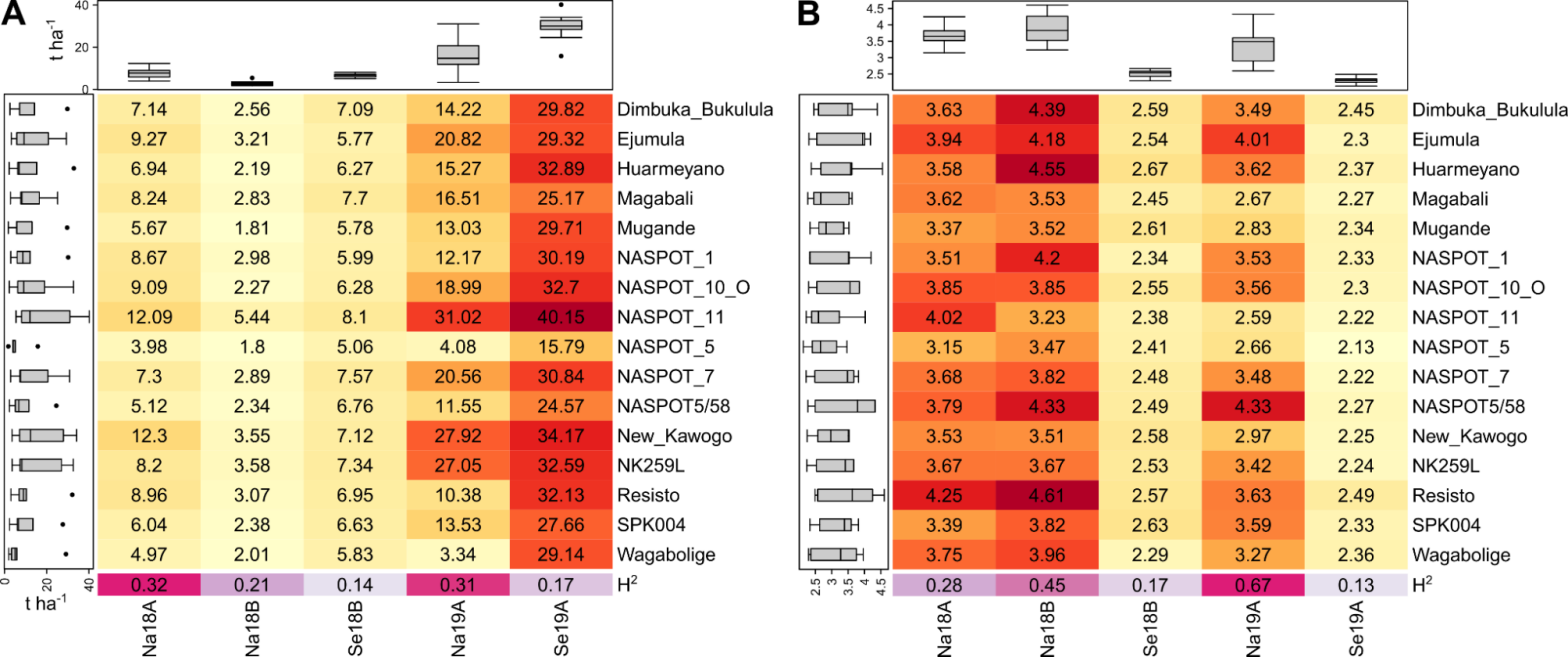
Within-environment best linear unbiased predictions (BLUPs) of (**A**) storage root yield (t ha^–1^), and (**B**) SPVD symptoms of 16 parents, and generalized measures of heritability (${H}^{2}$) for the population (progenies of 64 families and 16 parents) per environment in clonal plot trials. The box-plots on the left and top of the heatmaps depict the distribution of values per clone (left) and per environment (top). These heatmaps were created using the R package ComplexHeatmap^29^, version 2.20.0 (https://jokergoo.github.io/ComplexHeatmap-reference/book/).

Average storage root yield of parents ranged from 6.14 t ha^–1^ (‘NASPOT 5’) to 19.4 t ha^–1^ (‘NASPOT 11’), and SPVD symptom scores from 2.76 (‘NASPOT 5’) to 3.51 (‘Resisto’) across environments (Fig. 2). The comparison between the parents’ means within the same year and season shows that yield was higher in Serere than in Namulonge, where SVPD was more severe. Heritability ranged from 0.14 to 0.32 for storage root yield, and 0.13 to 0.67 for SPVD symptoms (Fig. 2).

### Tester’s identification

**Fig. 3:**
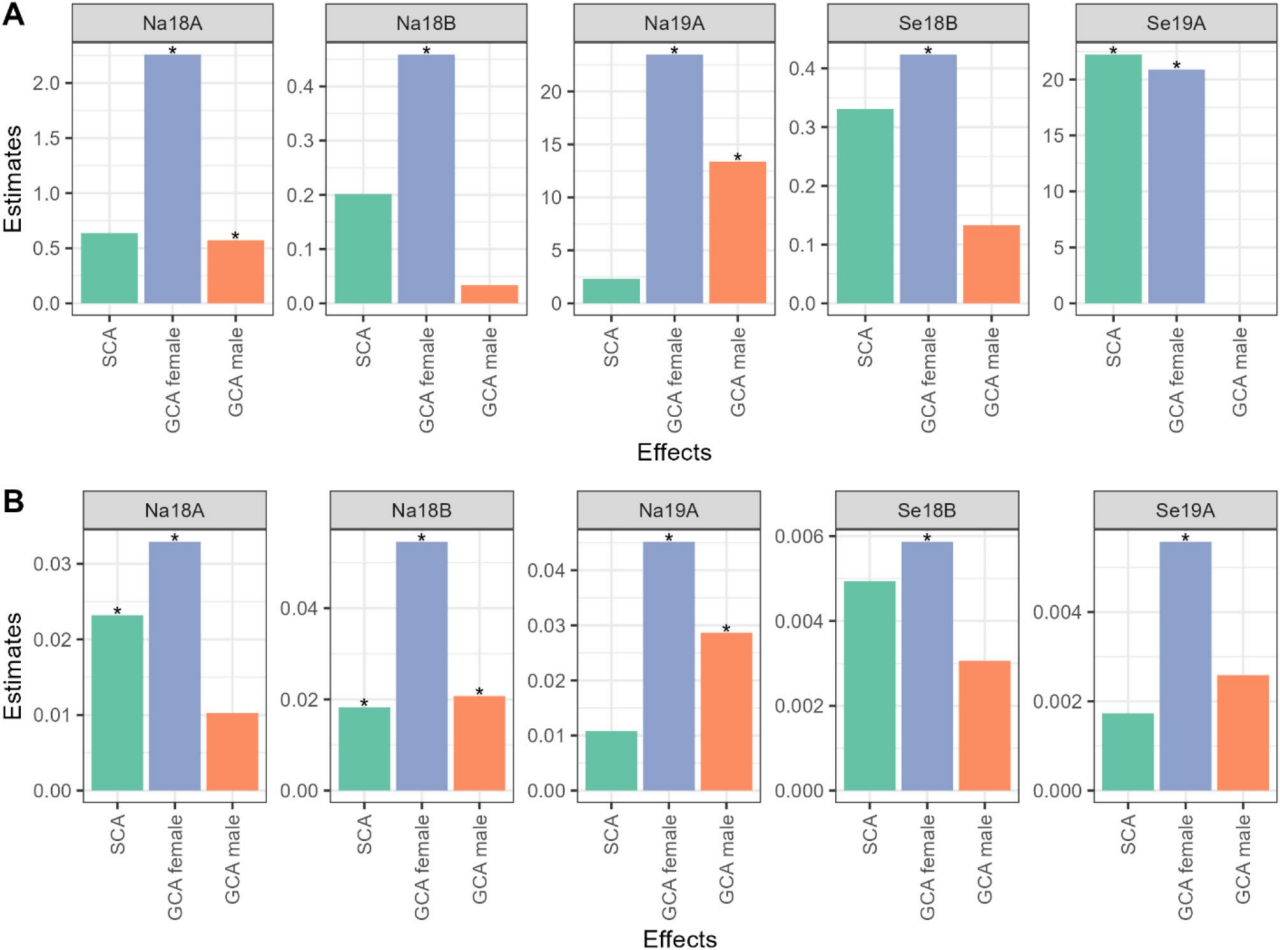
Genetic variance components for general combining ability (GCA) for 8 males, 8 females, and specific combining ability (SCA) for 8 × 8 crosses for (**A**) storage root yield (t ha^–1^), and (**B**) SPVD symptoms, in five environments in Uganda: Namulonge (Na) and Serere (Se) in seasons 2018 A, 2018 B, and 2019 A (Na18A, Na18B, Na19A, Se18B, Se19A; A = main rainy season, B = short rainy season). The asterisks on the top of each bar illustrate the effects’ significance, according to the likelihood ratio test. These plots were created using the R package ggplot2^30^, version 3.5.1 (https://ggplot2.tidyverse.org/); and stacked using the R package ggpubr^31^, version 0.6.0 (https://rpkgs.datanovia.com/ggpubr/).

GCA estimates had a wider range among the female parents than in the male parents in both traits (Fig. 3). ‘NK259L’ was the best male in both traits, with the highest GCA for storage root yield, and the lowest for SPVD symptoms, i.e. towards resistance. This coincidence did not happen between females: ‘NASPOT 11’ showed highest GCA for storage root yield, and ‘NASPOT 5’ the lowest for SPVD symptoms. Overall, the GCA of females and SCA represented the greatest part of the genetic variance in all environments. Females’ GCA effects were significant in all environments and traits, whilst the significance of males’ GCA and SCA effects varied per trait and environment (Fig. 3; Suppl. Table S2).

**Fig. 4:**
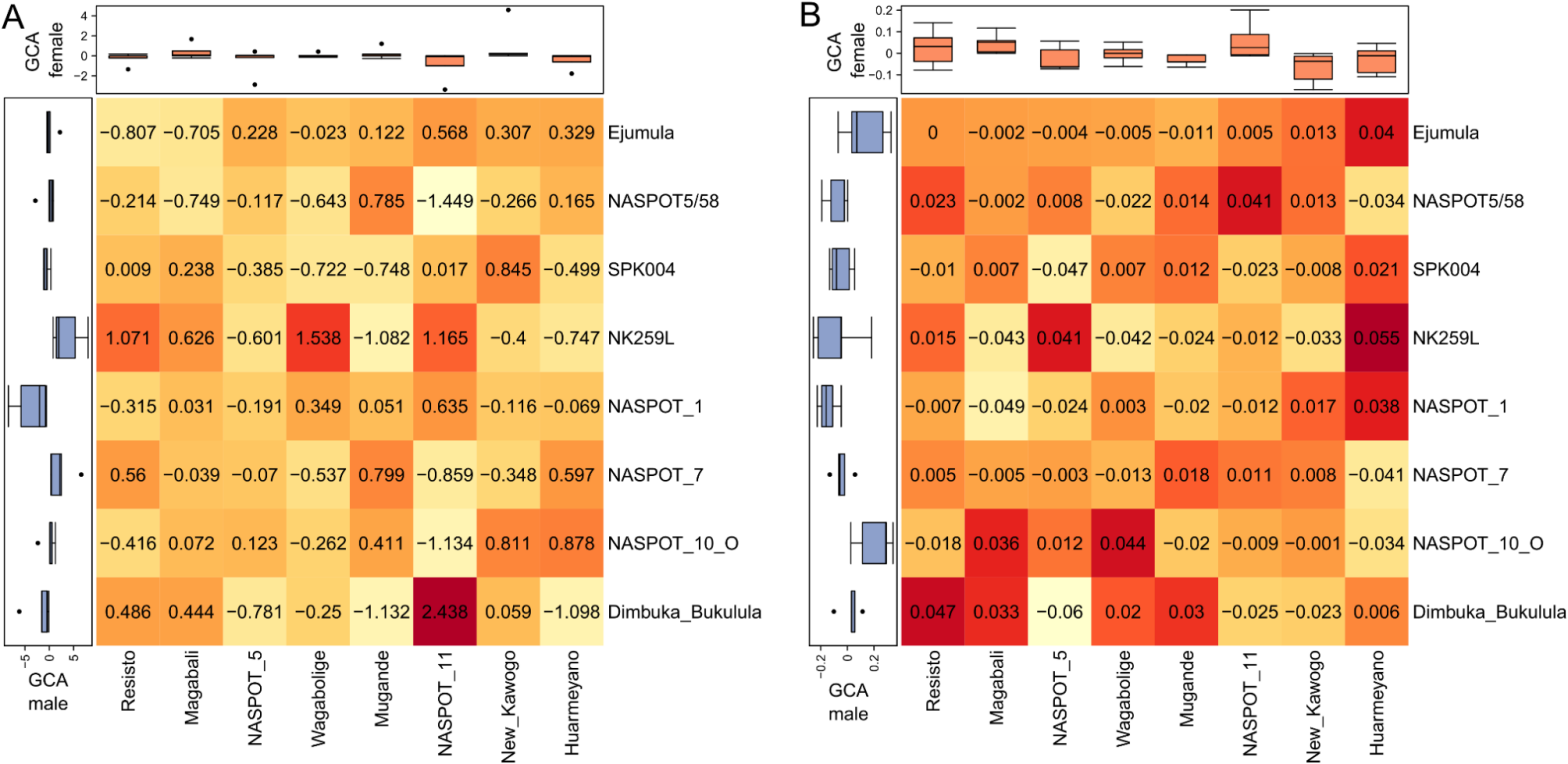
Average specific combining abilities (SCA) for (**A**) storage root yield (t ha^–1^), and (**B**) SPVD resistance between males from pool A (*x*-axis) and females from pool B (*y*-axis). The vertical and horizontal boxplots represent the general combining ability (GCA) of male and female parental clones, respectively. These heatmaps were created using the R package ComplexHeatmap^29^, version 2.20.0 (https://jokergoo.github.io/ComplexHeatmap-reference/book/).

The best crosses varied for each trait: ‘Dimbuka Bukulula’ × ‘NASPOT 11’ for storage root yield, and ‘NASPOT 1’ × ‘Magabali’ for SPVD resistance (Fig. 4). Note that the best male (‘NK259L’) was not part of the best cross in any trait. Still, its progenies featured among the top 10 crosses for both traits (three progenies for storage root yield and four progenies for SPVD symptoms). The best females for storage root yield and SPVD resistance, ‘NASPOT 11’ and ‘NASPOT 5’, respectively, featured in two crosses among the 10 top ones considering the ranking for the corresponding trait (Fig. 4).

**Fig. 5:**
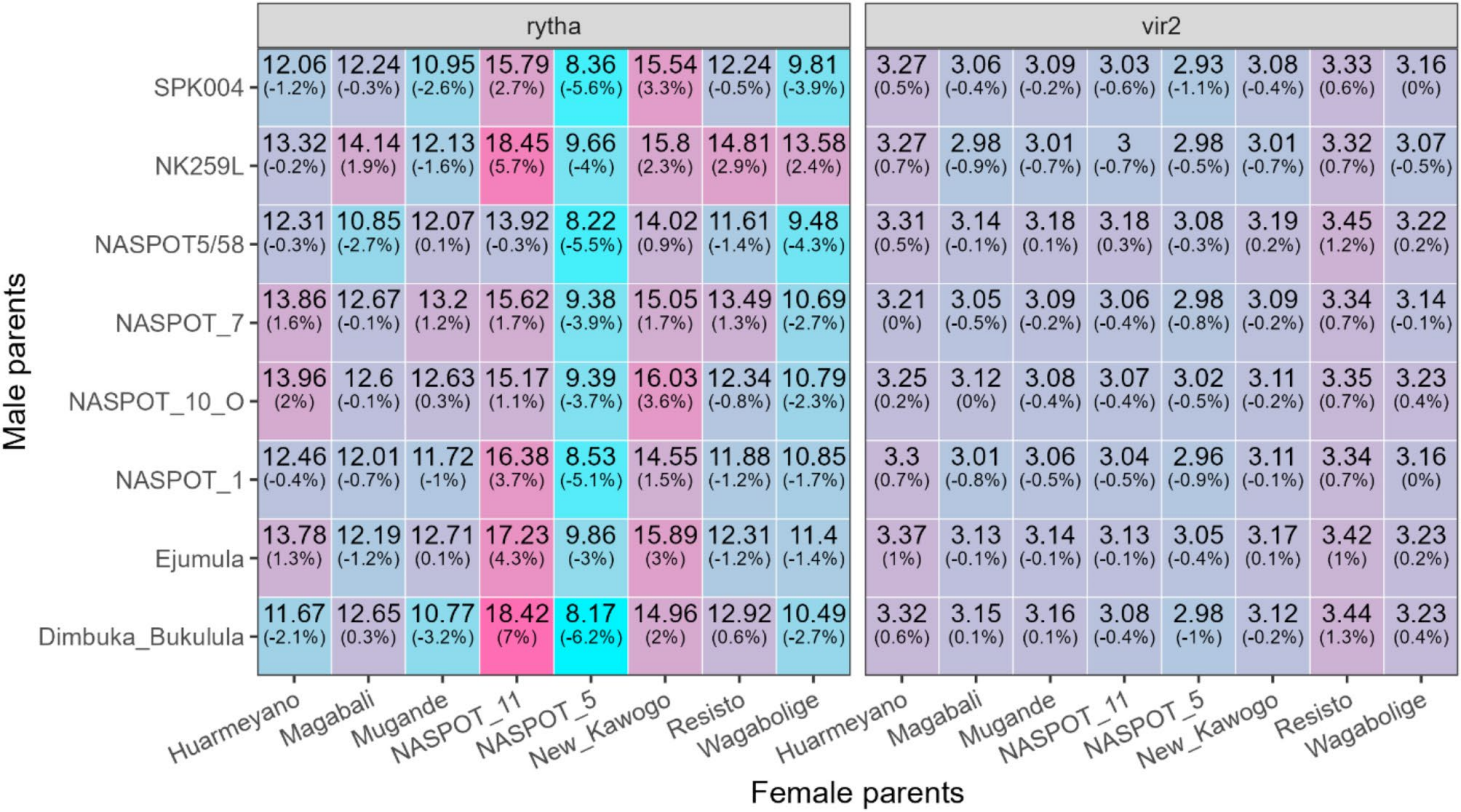
Predictions of storage root yield (t ha^–1^, rytha) and SPVD symptoms (vir2) of 64 families and heterosis increments in parentheses based on family prediction – mid-parent value evaluated in five environments in Uganda, Namulonge and Serere in seasons 2018 A, 2018 B, and 2019 A (A = first rainy season, B = second short rainy season). Shades of pink and blue cells indicate positive and negative heterosis increments, respectively. These plots were created using the R package ggplot2^30^, version 3.5.1 (https://ggplot2.tidyverse.org/).

Mid-parent heterosis estimates ranged from −6.2% (‘Dimbuka Bukulula’ × ‘NASPOT 5’) to 7.0% (‘Dimbuka Bukulula’ × ‘NASPOT 11’) for storage root yield, and from −1.1% (‘SPK004’ × ‘NASPOT 5’) to 1.3% (‘Bimbuka Bukulula’ × ‘Resisto’) for SPVD resistance (Fig. 5). For the top 1 female parent of each trait (‘NASPOT 11’ for storage root yield and ‘NASPOT 5’ for SPVD symptoms), most of the heterosis increments were positive (or negative, in the case of SPVD resistance), meaning that family predictions were higher than the mid-parent means. The exception was the cross between ‘NASPOT 11’ and ‘NASPOT5/58’ for storage root yield. Different from the female parents, top male parents did not generate families with only positive (or only negative) heterosis increments. Particularly, ‘NK259L’ generated heterosis increments ranging from −4.0 to 5.7% for storage root yield, and −0.7 to 0.7% for SPVD symptoms.

**Fig. 6:**
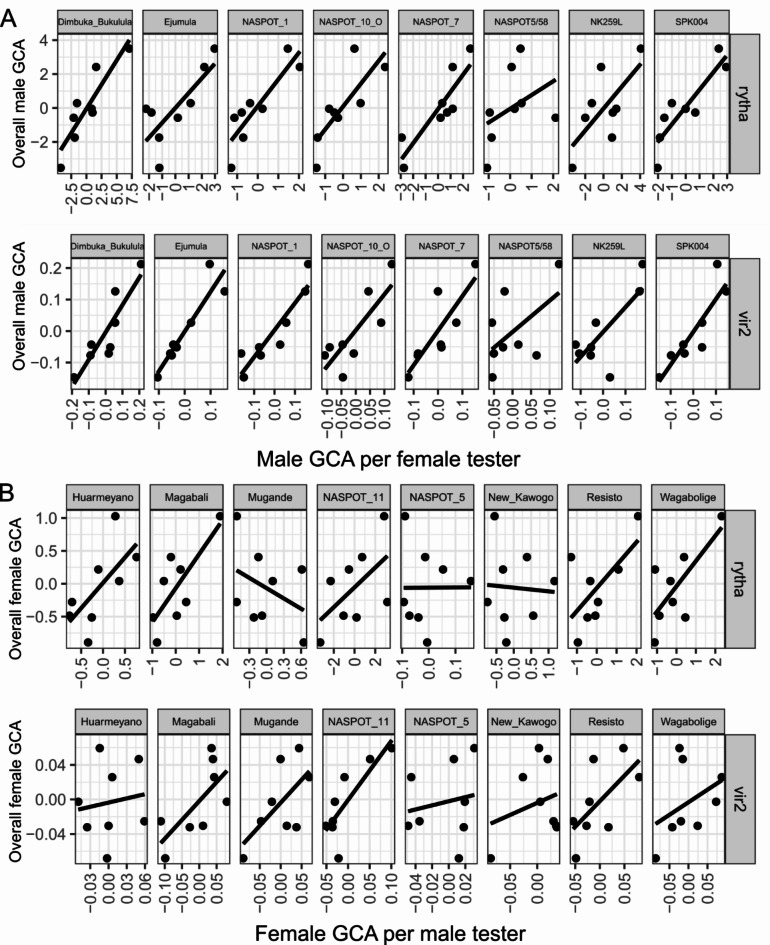
Scatter plots of the general combining ability (GCA) of the male (**A**) or female (**B**) parents regressed on the GCA estimates of the female or male parents, where each subset represents a male or female parent for storage root yield (t ha^–1^, rytha) and SPVD resistance (vir2). These plots were created using the R package ggplot2^30^, version 3.5.1 (https://ggplot2.tidyverse.org/); and stacked using the R package ggpubr^31^, version 0.6.0 (https://rpkgs.datanovia.com/ggpubr/).

The regression of male GCA estimates on each subset of female parents (Step 2 in Fig. 1) is not perfect (Fig. 6A), meaning that some males are better suited to serve as testers than others. The same goes for females (Fig. 6B). The correlations between the GCA estimates obtained in the second step and the GCA estimates obtained from the subset of each female or male ranged from −0.384 (‘Mugande’) to 0.893 (‘Dimbuka Bukulula’) for storage root yield, and from 0.139 (‘Huarmeyano’) to 0.928 (‘Ejumula’) for SPVD symptoms (Table 1).

The identification of testers (Step 3 in Fig. 1) combined information from the two previous steps as shown in Table 1. A parent was classified as a potential tester (‘yes’ in the column “Tester?” of Table 1) if presented with a positive (for rytha) or negative (for vir2) GCA, positive (for rytha) or negative (for vir2) average SCA across families, and correlation above 0.7. The selection of potential testers depends on the product profile and the breeding program’s objective. In single-trait analyses, ‘Ejumula’, ‘NASPOT 10 O’, and ‘NASPOT 7’ would be the selected to be testers for rytha; and ‘NASPOT 1’, ‘NASPOT 7’, ‘NK259L’, ‘SPK004’, and ‘NASPOT 11’ would be the most appropriate testers for vir2. The only tester for both traits would be ‘NASPOT 7’.

**Table 1:** Parents evaluated for their potential as testers for two traits: storage root yield (t ha^–1^, rytha) and SPVD symptoms (vir2). A parent is a potential tester if GCA > 0 for rytha or < 0 for vir2, average SCA across families > 0 for rytha or < 0 for vir2 (Step 1) and correlation (Corr.) > 0.70 (Step 2). Parents from the population (pop.) A were males, and from the population B were females.

Parent	Pop.	rytha				vir2			
		Step 1		Step 2	Step 3	Step 1		Step 2	Step 3
		GCA	SCA	Corr.	Tester?	GCA	SCA	Corr.	Tester?
Dimbuka Bukulula	A	− 0.278	0.021	0.893	No	0.026	0.004	0.884	No
Ejumula	A	0.406	0.002	0.767	Yes	0.047	0.004	0.928	No
NASPOT 1	A	− 0.513	0.047	0.876	No	− 0.025	− 0.007	0.918	Yes
NASPOT 10 O	A	0.041	0.06	0.803	Yes	− 0.003	0.001	0.81	No
NASPOT 7	A	0.217	0.013	0.885	Yes	− 0.032	− 0.002	0.839	Yes
NASPOT5/58	A	− 0.893	− 0.311	0.388	No	0.059	0.005	0.546	No
NK259L	A	1.027	0.196	0.683	No	− 0.068	− 0.005	0.761	Yes
SPK004	A	− 0.486	− 0.156	0.888	No	− 0.031	− 0.005	0.885	Yes
Huarmeyano	B	0.284	− 0.056	0.677	No	0.126	0.006	0.139	No
Magabali	B	− 0.271	− 0.01	0.765	No	− 0.071	− 0.003	0.692	No
Mugande	B	− 0.576	− 0.099	− 0.384	No	− 0.052	0	0.629	No
NASPOT 11	B	3.5	0.173	0.563	No	− 0.077	− 0.003	0.819	Yes
NASPOT 5	B	− 3.529	− 0.224	0.00357	No	− 0.147	− 0.01	0.174	No
New Kawogo	B	2.418	0.112	− 0.0588	No	− 0.043	− 0.002	0.262	No
Resisto	B	− 0.046	0.047	0.627	No	0.213	0.007	0.636	No
Wagabolige	B	− 1.745	− 0.069	0.72	No	0.027	− 0.001	0.386	No

## Discussion

### Performance of parents, genetic variance, and heritability

The mean performance of parents across environments was influenced by the exceptionally low storage root yields of the parents observed during the 2018 trials. Such low values could be attributed to the erratic rainfall patterns in Uganda [Suppl. Fig. S1]^32^. Although sweetpotato is adaptable to dry areas, drought usually leads to a significant decrease in storage root yield^33^. Furthermore, the stressful environment makes the plants more prone to diseases and pests. The higher storage root yield and their larger heritability in the main rainy growing season compared to the second growing season sheds light on the impact that abiotic factors have on yield in sweetpotato. Larger genotypic differences in high-yielding environments were also demonstrated by Swanckaert et al.^34^ and Grüneberg et al.^35^. Biotic and abiotic stress factors influencing storage root yield, include SPVD pressure, drought, and soil fertility^36^. The lower yield in Namulonge is accompanied by the higher incidence of SPVD symptoms in this location, regardless of season. SPVD pressure is very high in East Africa as discussed by Gibson and Kreuze^37^, and clonal evaluation in such environments can impact the genotypes’ performance. However, the SPVD pressure can also be an opportunity for breeders as long as they select genotypes that outperform their peers even under severe disease incidence.

The current study reported on storage root yield heterosis, which has the potential to gain from a hybrid breeding scheme as demonstrated by Grüneberg et al.^35^ in Peru. We also included SPVD resistance in the analyses, an economically important trait. Notably, when selecting parents for crossing blocks, other traits related to product quality (such as root color, shape, texture), vigor, and tolerance to abiotic stresses should be considered. The ultimate goal should be to direct crosses so that the offspring inherit favorable alleles for these traits.

The generalized measures of heritability varied for storage root yield and SPVD resistance. The $H^{2}$ estimates are important measures of resemblance between offspring and their parents^38^. Resemblance among clones was accessed by the pedigree combined with parental genomic information in our study, which allowed the estimation of additive genetic variances in each location. Our results indicate that the phenotypes are poor indicators of sweetpotato breeding values so the selection should not be based on phenotypic values on *per se* performance alone, and selection of parents (identification of good family makers) on their offspring performance is very important.

### General combining ability, specific combining ability, and mid-parent heterosis

GCA drives the selection of new sweetpotato parents in a RRS scheme. The genetic variance of GCA suggests that it is possible to select parents with high breeding values within each pool for future crosses^2^. Parents with a high GCA also had positive desirable SCA, making the selection of potential testers more straightforward. This would be useful to create crosses with a high genetic value for use as hybrids.

The mid-parent heterosis is assumed to be a good predictor of the additive gene expression^39^. Increased yields due to heterotic vigor can be captured in the SCA which includes all non-additive effects. Parents whose progenies show high mid-parent heterosis can also be candidates for having superior families if they show good performance on the evaluated traits. Improved storage root yield and SPVD symptoms of families can be explained by favorable allelic interactions at heterozygous loci or because deleterious and recessive alleles of parents are masked in families^2^. Recent studies have shown heterosis increments for sweetpotato hybrid populations^11,40^. Grüneberg et al.^11^ estimated heterosis increments for storage root yield in three orange-fleshed sweetpotato hybrid populations developed in Peru. The authors found population average heterosis increments of up to 43.5% and stated that population hybrid breeding is a tool to achieve remarkable genetic gains for sweetpotato root yields in the range of 81.5–132.4%. The advantage of this study was that it used applied breeding material on scale and three different populations aiming at four target product profiles, but only one complete RRS selection cycle. Diaz et al.^40^ investigated the genetic diversity of two South American orange-fleshed sweetpotato breeding populations (Jewel and Zapallo) for storage root yield and their hybrid population heterosis increments. The authors estimated average heterosis increments of 21.8% and that heterosis increments contributed considerably to hybrid performance and nearly as much as the per-se performance of parents so that both should be used for selecting sweetpotato parental selection (per-se performance and heterosis by using offspring information). These two populations were considered useful for studying the efficiency of RRS in sweetpotato population hybrid breeding realized later^11^.

Although positive (or negative, for SPVD symptoms) heterosis was observed, the estimates were not high, mainly for SPVD symptoms, likely due to the bias SPVD has on heterosis increments for storage root yield or the lack of true genetic separation among parents from the two pools performed using SSR as recently observed via genome-wide single-nucleotide polymorphisms (SNPs) (Suppl. Fig. S3). Akinwale^41^ pointed out that the usage of molecular markers should be preferred when defining heterotic pools due to their precision since they are minimally influenced by environmental factors. The extensive use of molecular markers has allowed a clear definition of heterotic pools in tropical^42^ as well as temperate^43^ maize germplasm, leading to expressive heterosis exploitation in inter-pool crosses in the crop^41^. A positive correlation between genetic distance and heterosis for lint percentage and micronaire in upland cotton (*Gossypium hirsutum* L.) was observed by Geng et al.^44^. Yet, some studies have shown no association between genetic distance and heterosis^45^. Geng et al.^44^ suggested that the association depends upon the germplasm, type of molecular marker, genome coverage, and genome region of the molecular marker. SNP-based clustering analysis revealed that MDP parents from pools A and B do not show clear separation^16^. The authors stated that clustering the MDP parents into two different pools based on microsatellite markers^18^ was inconsistent with the clustering of the same accessions based on > 1 M SNPs. Despite initial incorrect pool allocation, the population development pipeline (within pool cross and selection) will likely lead to divergence between pools and increase heterosis as observed by^40^.

### Tester’s identification

The ideal procedure for tester selection is still a challenge in breeding programs aiming at hybrid development. It is fundamental that a tester sufficiently correct shows the genetic merit of the genotype being crossed^4^. Once testers are selected, topcrosses using the selected testers can overcome the need for large offspring evaluations and the chance of underrepresentation of parents due to incompatibilities in sweetpotato crosses. This optimization can be applied either to an RRS or a one-pool strategy. Having good GCA estimates demands very large phenotyping efforts where offspring clones with known pedigree need to be generated and evaluated in multiple environments. Reports on combining ability in sweetpotato used 10 to 56 families from 4 to 30 full-sibs per family, resulting in an evaluation of 40 to 150 offspring clones^8,46–48^. This study has, for the first time, developed a procedure to identify testers in sweetpotato based on GCA, SCA, and the ability to estimate GCA on a subset of parents of interest. The current three-step approach to identifying potential testers involves selecting parents and crosses showing positive GCA and SCA effects (Step 1), respectively, and the correlation between the GCA obtained in Step 1 and the GCA estimates from the subset of parent $i$ (Step 2).

Positive GCA and SCA effects are of interest since we seek to find clones and crosses contributing to increasing the yield mean in the breeding program. The same idea is valid for SPVD symptoms, but in the other direction: GCA and SCA should be negative. The correlation criterion adopted in Step 2 is advised to be higher than 0.7 as a way to guarantee proper GCA assessment. Testers are important in hybrid breeding schemes because they can estimate the GCA in a larger population of parents from an opposing pool, allowing much larger selection intensity. Moreover, they can also enhance population parameters and maximize genetic gain in a one-pool breeding scheme.

## Concluding remarks

The usefulness of the proposed three-step approach goes beyond thresholds and selection direction, which depends on the trait. The selection of a potential tester based on different selection steps possibly surpasses the selection based on GCA or SCA effects alone. Using the proposed methodology, we identified three testers for storage root yield and five testers for SPVD resistance. Additionally, we found one tester (‘NASPOT 7’) with dual aptitude, being appropriate for both assessed traits.

## Electronic supplementary material

Below is the link to the electronic supplementary material.

Supplementary Material 1

Supplementary Material 2

## Data Availability

Data is provided within the manuscript or supplementary information files.
