# Supplementary material for: Tester selection for combining ability estimation of storage root yield and sweetpotato virus disease in sweetpotato breeding
Source: Sci Rep. 2025 Feb 10;15:4951. doi: 10.1038/s41598-025-88609-w (PMC11811066; doi:10.1038/s41598-025-88609-w)
Supplement: Supplementary file 2 — Supplementary Material 2 [file 41598_2025_88609_MOESM2_ESM.docx]

**Supplementary Information (SI)**

**Tester selection for combining ability estimation of storage root yield in sweetpotato breeding**

Jolien Swanckaert^1†^ (0000-0002-3694-4834), Iara Gonçalves dos Santos^2†^ (0000-0002-5910-6075), Saulo F. S. Chaves^2^, Reuben Ssali^1^, Robert O.M. Mwanga^1^, Camila Ferreira Azevedo^2^, Thiago O. Mendes de Paula^3^, Bert De Boeck^4^, Raul Eyzaguirre^4^, Mercy Kitavi^5^, Dorcus C. Gemenet^6^, Maria Andrade^7^, Wolfgang J. Grüneberg^4^, Hannele Lindqvist-Kreuze^4^, G. Craig Yencho^8^, Hugo Campos^4^, Guilherme da Silva Pereira^2^*

^1^ International Potato Center (CIP), PO Box 22274, Kampala, Uganda; ^2^ Federal University of Viçosa (UFV), Viçosa, Brazil; ^3^ International Potato Center (CIP), ILRI Campus, Nairobi, Kenya; ^4^ International Potato Center (CIP), Av. La Molina 1895, Lima, Peru; ^5^ University of Georgia (UGA), Athens, GA, USA ^6^ CGIAR Excellence in Breeding (EiB) Platform, CIMMYT-Nairobi, Kenya; ^7^ International Potato Center (CIP), Maputo, Mozambique; ^8^ North Carolina State University (NCSU), Raleigh, NC, USA

^†^ These authors contributed equally to this work.

Corresponding author: g.pereira@ufv.br

**Table S1**. Description of parents of the Mwanga diversity panel (MDP).

| Name | CIP code | Cultivar type⸸ | Cultivar origin | Root shape | Flesh colour^#^ | Skin colour^#^ | Plant type | Group |
| --- | --- | --- | --- | --- | --- | --- | --- | --- |
| Dimbuka-Bukulula | CIP443752 | FV | Uganda | Long irregular | C | C | Spreading | Male |
| Ejumula | CIP443750 | FV | Uganda | Long irregular | DO | C | Spreading | Male |
| Huarmeyano | CIP420020 | FV | Peru | Round | C | O | Extremely spreading | Female |
| Magabali | *NA* | FV | Uganda | Long elliptic | C | C |  | Female |
| Mugande | CIP440163 | FV | Rwanda | Long elliptic | W | PR | Semi-erect | Female |
| NASPOT 1 | CIP191133.1 | MV | Uganda | Obovate | Y | C | Spreading | Male |
| NASPOT 10 O | CIP100200.4 | MV | Uganda | Long irregular | O | PR | Semi-erect | Male |
| NASPOT 11 | CIP100201.1 | MV | Uganda | Long elliptic | C | PR | Erect | Female |
| NASPOT 5 | CIP191133.5 | MV | Uganda | Round elliptic | O | C | Spreading | Female |
| NASPOT 7 | CIP100200.1 | MV | Uganda | Ovate | O | PR | Semi-erect | Male |
| NASPOT5/58 | *NA* | BL | Uganda |  | O | O | Spreading | Male |
| New Kawogo | CIP441743 | FV | Uganda | Obovate | W | PR | Spreading | Female |
| NK259L | *NA* | BL | Uganda | Obovate | C | PR | Spreading | Male |
| Resisto | CIP440001 | MV | USA | Ovate | DO | BO | Semi-erect | Female |
| SPK004 | CIP441768 | FV | Kenya | Long irregular | PO | P | Spreading | Male |
| Wagabolige | CIP440167 | FV | Uganda | Round | C | C | Spreading | Female |
| ⸸ BL, breeding line; FV, farmer variety; MV, modern variety, *NA*, not applicable. | | | | | | | | |
| ^#^ BO, brownish orange; C, cream; DO, dark orange; O, orange; P, pink; PO, pale orange; PR, purple red; W, white; Y, yellow | | | | | | | | |

**Table S2**. Variance components of the GCA (female and male) and SCA effects, and their respective likelihood ratio test results: values followed by an asterisk (*) depict significant effects at a 5% probability and one degree of freedom in the chi-square table.

| Environment | Storage root yield | | | Virus resistance | | |
| --- | --- | --- | --- | --- | --- | --- |
|  | GCA | | SCA | GCA | | SCA |
|  | Female | Male |  | Female | Male |  |
| Na18A | 2.260* | 0.573 | 0.637* | 0.033* | 0.010 | 0.023* |
| Na18B | 0.459* | 0.033 | 0.202 | 0.055* | 0.021* | 0.018* |
| Na19A | 28.093* | 13.394* | 2.306 | 0.045* | 0.029* | 0.011 |
| Se18B | 0.424* | 0.133 | 0.331 | 0.006* | 0.003 | 0.005 |
| Se19A | 20.882* | - | 22.231* | 0.006* | 0.003 | 0.001 |

Na18A = Namulonge 2018 first rainy season; Na18B = Namulonge 2018 second short rainy season Se18B = Serere 2018 second short rainy season; Se19A = Serere 2019 first rainy season


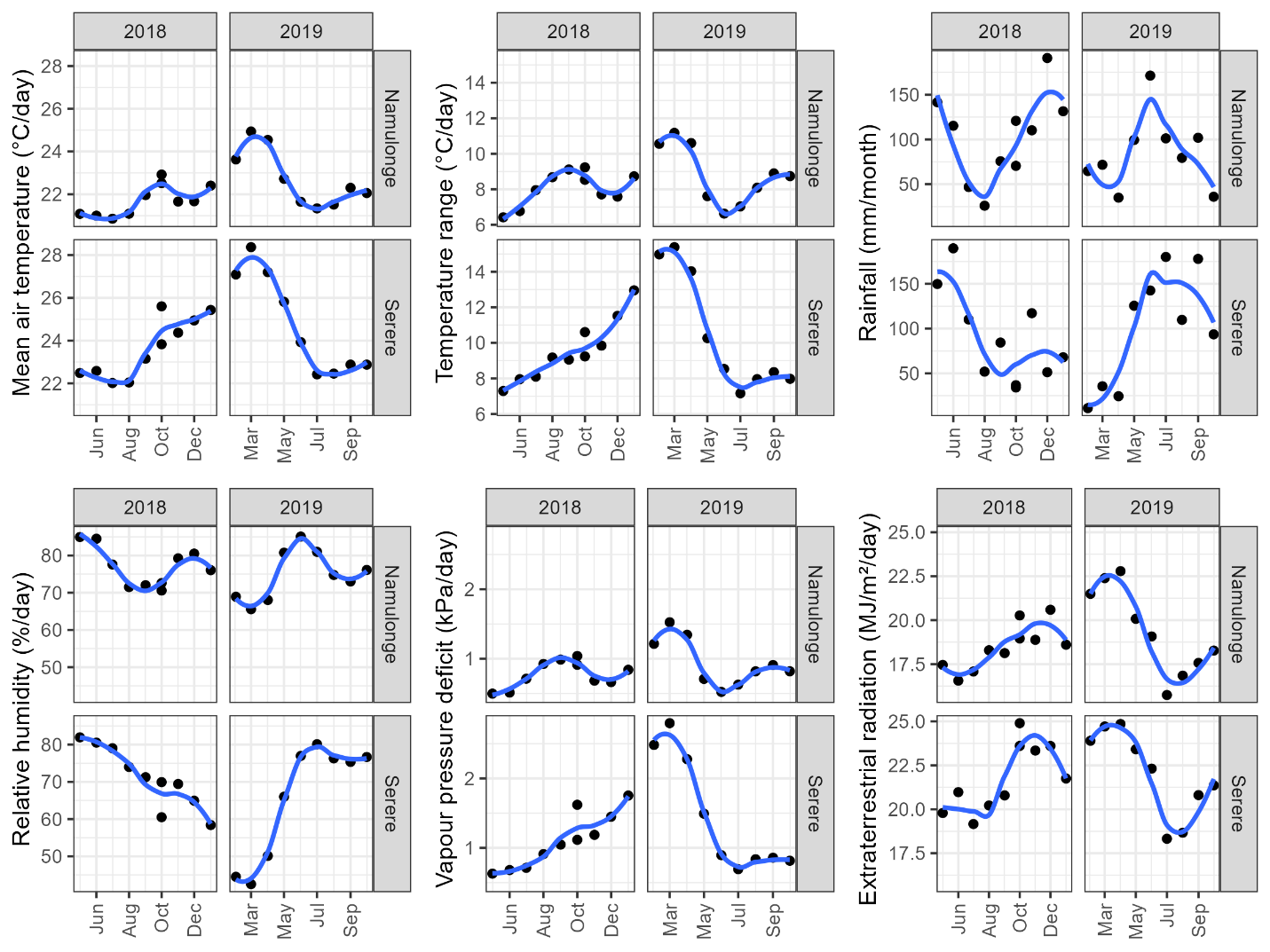


**Fig. S1.** Weather information from the two studied locations during the months of the trial, in 2018 and 2019. Rainfall is registered as the monthly total whereas the other variables are given as monthly averages. The smooth lines have no statistical purposes and were plotted just to guide interpretation regarding the trends. These plots were created using the R package ggplot2 [1], version 3.5.1 (<https://ggplot2.tidyverse.org/>); and stacked using the R package ggpubr [2], version 0.6.0 (<https://rpkgs.datanovia.com/ggpubr/>)


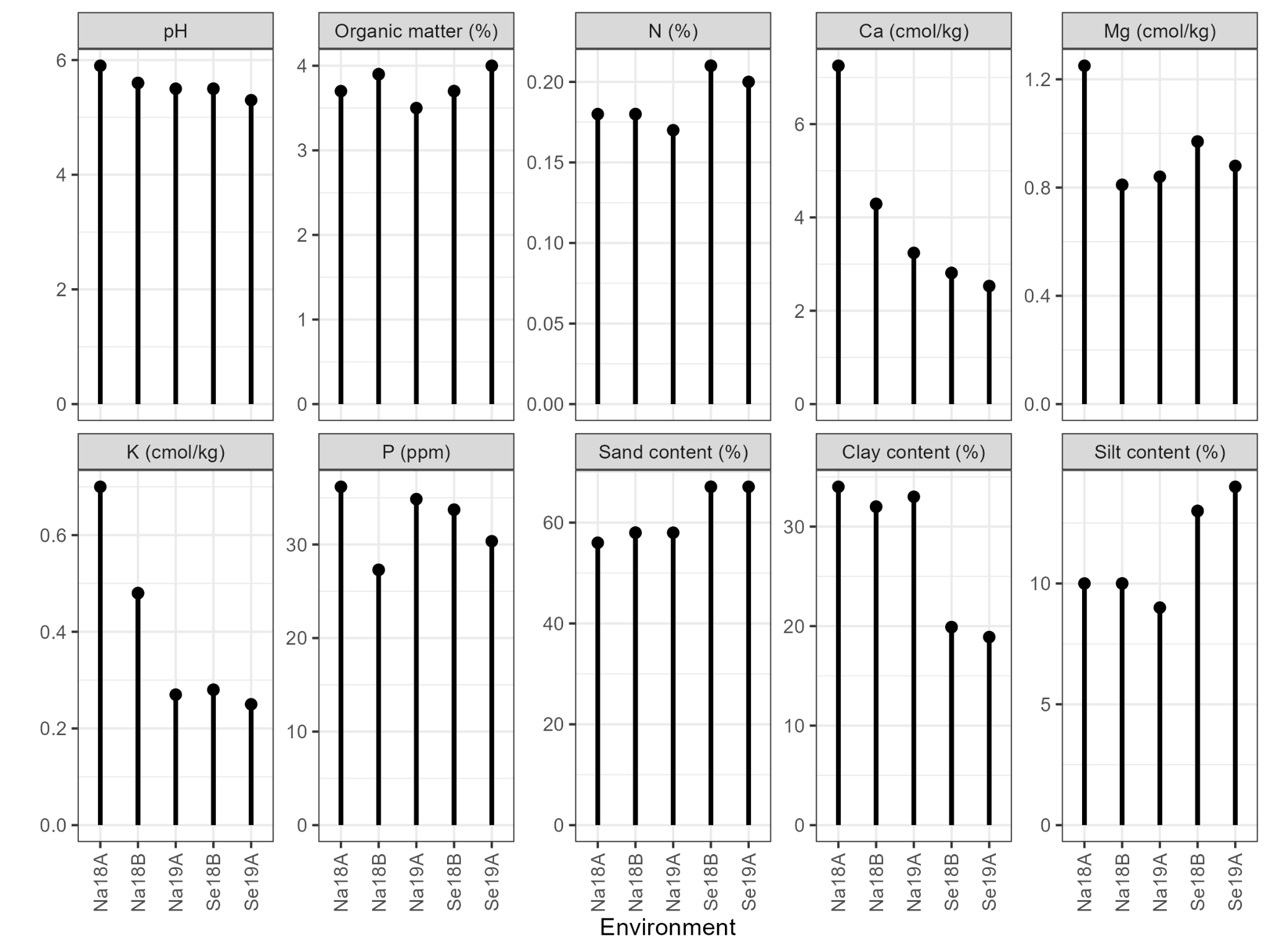


**Fig. S2.** Soil information for soil samples from the two locations and seasons where the trial was conducted, Namulonge 2018, 2019, A (main rainy season) and B short rainy season) (Na18A, Na18B, Na19A) and Serere 2018, 2019 A and B (Se18A, Se19, Se19A); soil pH; N, nitrogen; Ca, calcium; Mg, magnesium; K, potassium; P, phosphorus; %, percent. These plots were created using the R package ggplot2 [1], version 3.5.1 (<https://ggplot2.tidyverse.org/>); and stacked using the R package ggpubr [2], version 0.6.0 (<https://rpkgs.datanovia.com/ggpubr/>)


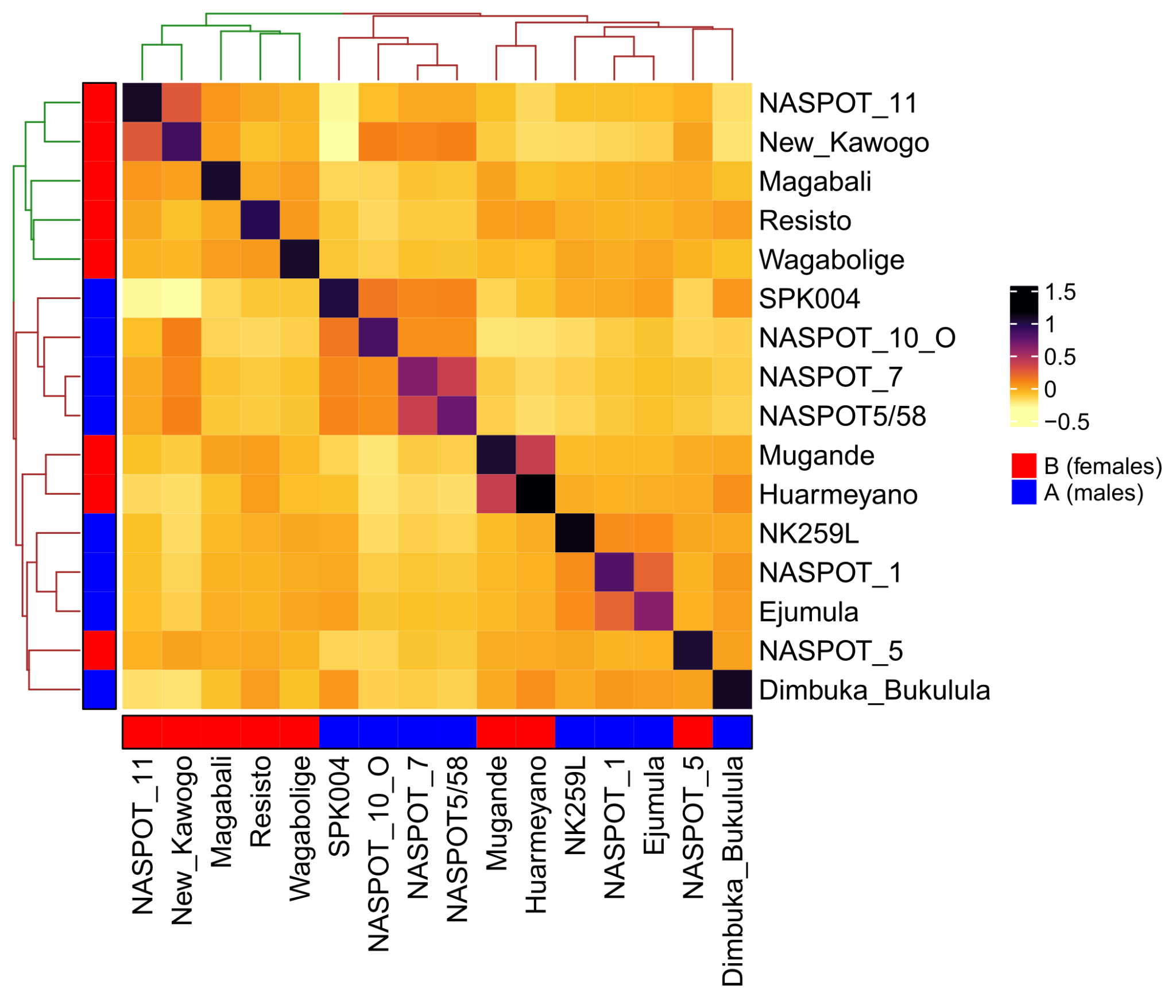


**Fig. S3.** Clustering of the 16 parents of the Mwanga diversity panel (MDP) based on the genomic relationship matrix ($G$) estimated from >1M single nucleotide polymorphisms (SNPs). This heatmap was created using the R package ComplexHeatmap [3], version 2.20.0 (<https://jokergoo.github.io/ComplexHeatmap-reference/book/>)

**References**

1. Wickham, H. *Ggplot2: Elegant Graphics for Data Analysis*. (Springer, Cham, 2016).

2. Kassambara, A. ggpubr: ggplot2 Based Publication Ready Plots. (2023).

3. Gu, Z. Complex heatmap visualization. *iMeta* **1**, e43 (2022).
